# Occupational Autonomy and Later Life Cognitive Change: The Moderating Role of Education

**DOI:** 10.1093/geroni/igaf122.1820

**Published:** 2025-12-31

**Authors:** Katy Cao, Dawn Carr

**Affiliations:** Florida State University, Tallahassee, Florida, United States; Florida State University, Tallahassee, Florida, United States

## Abstract

As a component of enriching occupational environments, work autonomy has been associated with workers’ better physical and mental health. However, few longitudinal studies have connected autonomous work with later-life cognition. Furthermore, although education selects people into different career paths, few studies examined whether the cognitive health impact of autonomous work depends on education. To address these gaps, we ask the following research question: Is the association between autonomous work and later-life cognitive maintenance robust across different levels of education? Using the longitudinal Health and Retirement Study and Occupational Information Network (O*NET) linked dataset, we examine participants’ 8-year cognitive change associated with occupational autonomy in mid-life (age 51-60), N = 4179. Interaction effects were tested using Ordinary Least Squares (OLS) regression with robust standard error. Five occupational autonomy measures were selected to comprehensively evaluate different dimensions of autonomy: (1) freedom to make decisions, (2) frequency of decision-making, (3) unstructured work, (4) schedule regularity, and (5) work value of independence. Findings from OLS and post-estimation Wald tests show that education significantly moderates the association between cognitive maintenance and three occupational autonomy measures: Freedom of decision-making (β = 0.05, p < 0.01), unstructured work (β = 0.05, p < 0.01), and work value of independence (β = 0.02, p < 0.10). A margins plot shows that freedom to make decisions was positively associated with cognitive maintenance among people with 14 or more years of education but not among those without. Results imply that enhancing the employment quality among individuals with less education calls for a multiple-dimensional approach, including but not limited to improving their occupational autonomy.

